# Validation of digital microscopy: Review of validation methods and sources of bias

**DOI:** 10.1177/03009858211040476

**Published:** 2021-08-26

**Authors:** Christof A. Bertram, Nikolas Stathonikos, Taryn A. Donovan, Alexander Bartel, Andrea Fuchs-Baumgartinger, Karoline Lipnik, Paul J. van Diest, Federico Bonsembiante, Robert Klopfleisch

**Affiliations:** 1 University of Veterinary Medicine, Vienna, Austria; 2 Freie Universität Berlin, Berlin, Germany; 3 University Medical Centre Utrecht, Utrecht, the Netherlands; 4 Animal Medical Center, New York, NY, USA; 5 University of Padova, Legnaro, Padua, Italy

**Keywords:** accuracy, concordance rate, digital pathology, digital microscopy, noninferiority, review, study design, validation, virtual microscopy, whole-slide images

## Abstract

Digital microscopy (DM) is increasingly replacing traditional light microscopy (LM) for performing routine diagnostic and research work in human and veterinary pathology. The DM workflow encompasses specimen preparation, whole-slide image acquisition, slide retrieval, and the workstation, each of which has the potential (depending on the technical parameters) to introduce limitations and artifacts into microscopic examination by pathologists. Performing validation studies according to guidelines established in human pathology ensures that the best-practice approaches for patient care are not deteriorated by implementing DM. Whereas current publications on validation studies suggest an overall high reliability of DM, each laboratory is encouraged to perform an individual validation study to ensure that the DM workflow performs as expected in the respective clinical or research environment. With the exception of validation guidelines developed by the College of American Pathologists in 2013 and its update in 2021, there is no current review of the application of methods fundamental to validation. We highlight that there is high methodological variation between published validation studies, each having advantages and limitations. The diagnostic concordance rate between DM and LM is the most relevant outcome measure, which is influenced (regardless of the viewing modality used) by different sources of bias including complexity of the cases examined, diagnostic experience of the study pathologists, and case recall. Here, we review 3 general study designs used for previous publications on DM validation as well as different approaches for avoiding bias.

Digital microscopy (DM) (as opposed to conventional light microscopy [LM]) describes viewing of digitized microscopic images at a computer workstation.^18,79^ Rapid digitization of entire glass slides (producing whole-slide images [WSI]) by whole-slide scanners has advanced digital pathology such that DM is feasible for diagnostic service in larger laboratories with high caseloads.^13,64–67^ Large-capacity whole-slide scanners allow routine acquisition of WSI at high resolution (unit: microns per pixel), which is the precondition required for pathologists to generate reliable diagnoses. Nevertheless, WSI are “only” a duplicate of glass slides with default scan parameters and possible artifacts, which has led to skepticism among pathologists regarding the diagnostic performance of DM. Those concerns are justified as there are essential differences between DM and LM in the way tissue sections are presented to and evaluated by pathologists. It is imperative to prove that interpretations of the WSI, that is, the obtained diagnoses, are overall at least noninferior or equivalent to LM.^17,56^ Regardless of these concerns, whole-slide imaging and DM has been fostered due to improvements of efficiency, management and economics of the laboratory workflow, possibility of easy remote consultation, improved pathologist work flexibility (including off-site case reading), and ergonomics.^13,18,64,67^ Notably, in challenging on-site staffing situations, such as the COVID-19 pandemic, DM may be an important tool to keep histology workflows running smoothly.^66^ Furthermore, digital slides have the capability of being analyzed by automated image analysis software.^18,48^ Thus, WSIs might provide an advantage in assisting the pathologist (computer-assisted diagnosis) which could further improve diagnostic accuracy and reproducibility as well as diagnostic efficiency in the future.^11,47^ The number of human and veterinary laboratories that have implemented this digital pathology technology is increasing. A generation of pathologists that switches to DM faces the challenge of ensuring an adequate diagnostic performance of the new DM workflow. Validation studies are one crucial step in overcoming these challenges.

For this article, we have reviewed published validation studies and summarized the different methods used. Our intent is not to give recommendations for any specific method or requirement. Instead, this review article may provide guidance when selecting a suitable validation method, taking into consideration the intended objective and possible sources of bias for each individual laboratory.

## Why Validate DM?

LM is used traditionally by pathologists to assess sections of processed tissues and it is historically considered the best practice (“gold standard”) for microscopic diagnosis of tissue changes. If it is to be replaced by DM as a slide viewing modality for routine primary diagnostics in veterinary laboratories, it must be ensured that diagnostic aptitude and consequently patient care is not compromised. By substituting LM with DM, we now risk introducing a new set of limitations and artifacts. For example, does the color representation of WSI change the quantitative interpretation of color intensity and HUE value of special stains (such as quantification of hemosiderin concentration with a special stain for iron^44^ or quantification of copper concentration in liver sections with rhodanine stain) or immunolabeling (including cutoffs for immunopositive and immunonegative)?^77^ Or, is the scan resolution sufficient for examination of subtle patterns in histology specimens? Is a single focus layer scan sufficient for cytologic specimens of fine needle aspirations and body fluids? Validation provides answers to those questions. It describes the ongoing process of establishing and documenting scientifically sound evidence that the technology performs as expected for the intended use.^56^ The main objective of a validation study is to ensure that DM (defined as the test modality) can be used as reliably as LM (defined as the “gold standard” or reference modality) for rendering a specific diagnosis. Depending on the study design used (see below), either high concordance, equivalency/superiority, or noninferiority between the 2 modalities are tested.

There are 3 contexts in which DM validation can be performed: vendor-driven, academic, and clinical studies.^36^ The objective of vendor-driven studies is to obtain clearance from regulatory agencies in order to enable vendors to market their devices for a certain use and to supply potential buyers with meaningful information about their system.^36^ For example, in human pathology, Royal Philips and Leica Biosystems have received approval from the US Food and Drug Administration to market their Philips IntelliSite Pathology Solution and Aperio AT2 DX System, respectively, for primary diagnostic use in a clinical setting.^30,32^

The goal of academic validation studies (published in peer-reviewed journal articles) is to examine general feasibility/applicability and limitations of DM.^36^ These studies are encouraged for virtually all pathology applications (formalin-fixed tissue sections, fine-needle-aspiration cytology, standard stains, special stains, immunohistochemical labeling, etc), subspecialties (organ systems, diagnosis, grading, finding/counting small objects, etc), and DM workflow parameters (WSI scanner types, scan resolution, z-stacking, monitor characteristics, etc). However, it has been emphasized that these parameters cannot necessarily be extrapolated between laboratories due to the heterogeneous study protocols and individual laboratory environment.^36^

A clinical validation study is done to evaluate, document, and approve performance of a DM workflow in a specific laboratory environment for each pathology application (histology with hematoxylin and eosin stain [HE], immunohistochemistry, etc).^36^ This intends to ensure that the combination of technology (hardware and software) in the specific laboratory environment is reliable for the intended everyday clinical setting.^29,56^ In human pathology, clinical validation of DM for primary diagnostic work is required for each laboratory that initiates a transition from LM to DM or undertakes “significant” changes to the workflow (eg, changing the type of whole-slide scanner).^29,56^ It is, however, not necessary to validate each pathology subspecialty that is going to be diagnosed digitally or each pathologist that is going to use DM.^56^ For veterinary pathology, there is currently no consensus as to whether a validation study in each laboratory is required. Also, there is limited information on validation practices for the (nonregulated and regulated) pre-/nonclinical environment in research and toxicologic pathology.^46,61^

## What Should Be Validated?

**Figure 1.**
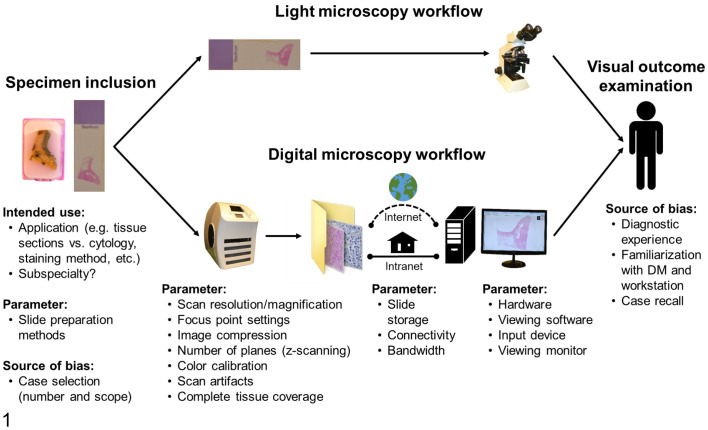
Light microscopy and digital microscopy workflow for a validation study, including associated “parameters” (technical aspects of the digital microscopy workflow that can be optimized if needed) and source of bias (factors that may influence light and digital microscopy). This scheme is modified from Bertram et al.^18^

Clinical validation studies are encouraged for each laboratory using DM and each intended clinical application of DM (such as evaluation of routine histologic sections, immunohistochemical specimens and cytology slides) closely emulating the “real life” environment.^29,56^ Implementation and stand-alone testing of individual technical components occurs before the validation study.^64,67^ Validation studies should encompass the entire digital microscopy workflow,^29,56^ which comprises slide preparation, “whole-slide imaging pixel pathway” (WSI acquisition, WSI storage and retrieval, workstation; see Bertram et al^18^ for more details) and visual outcome assessment (diagnosis) by a pathologist (Fig. 1). The individual steps and technical equipment of the DM workflow have “parameters” that need to be preset (based on test results and experience) for the validation study and intended use. Some of these parameters, such as scan magnification, are a tradeoff between ideal image quality and economic factors.^13^ The validation process primarily determines whether the preset parameters as a whole are acceptable for visual slide evaluation by pathologists or whether some parameters need to be optimized. Scan magnification and associated resolution is such a parameter. For example, a laboratory may have previously decided to routinely scan at low magnification (200×), but discover during the validation process that identification of mitotic figures requires higher image resolution. As a consequence, the laboratory might decide to optimize the parameter “scan magnification” from 200× to 400×for tumor cases.

Aside from those workflow parameters, there are some expected sources of bias that can significantly influence the outcome assessment of validation studies, but are not caused by the technical equipment of the DM workflow. The most relevant source of bias of the DM and LM workflow are the cases selected for the study and the individual pathologist(s) that are reviewing the cases (Fig. 1). Trained pathologists have very high, but not perfect, visual and cognitive abilities to obtain histologic diagnoses that might result in small inter- and intraobserver discrepancies for both viewing modalities. From this point of view, we do not consider the individual pathologist as a component that needs to be assessed in the context of a validating the DM workflow, as was done by Buck et al.^24^ However, interobserver agreement might be another aspect of quality control of certified medical laboratories (eg, ISO 15189), which can potentially be validated simultaneously with DM depending on the study design (see below). Pantanowitz et al^56^ highlight that validation of each individual pathologist for the use of DM is not necessary, however, including multiple study pathologists can account for individual preferences when considering the different viewing modalities.^17^ It is considered essential that each pathologist is trained prior to the use of DM in order to cope with the new technology appropriately and efficiently.^29,56^ Sources of bias should be mitigated as much as possible while obtaining and interpreting results^27^ and are discussed in the following sections.

## How Can DM Be Validated?

This section reviews the published “materials and methods” from validation studies. The appropriate magnitude of a validation study greatly depends on the individual goals, intended use, possible risks for patient care, available financial and labor/staff resources, expected cost/benefit ratio, and previous validation results. For example, if a routine DM workflow is to be implemented for the first time in a laboratory, then a more extensive validation study with a high number of cases and study pathologists as well as a more complex study design (see below) may be desired. Alternatively, if a well-implemented DM workflow must be nominally optimized or an additional application added, then a more simplistic approach may be sufficient. Since we consider a universal “one-size-fits-all” study method to be untenable, we provide an overview of multiple possible methods, which are also summarized in Supplemental Table S1. Guidelines on minimum requirements for a clinical validation study have been published by the College of American Pathologists in 2013^56^ and in 2021.^29^ Definitions for some relevant terms are provided in Table 1.

**Table 1.** Definitions of terms used in validation studies.

Term	Definition
Accuracy	Agreement between a study diagnosis and the ground truth diagnosis.
Concordance	Agreement between 2 study diagnoses, typically comparing LM versus DM diagnoses of the same case read by the same pathologist (intraobserver concordance).
Concordance rate	$\mathrm{Concordance rate}(\%)=\frac{\mathrm{concordant diagnoses}}{\mathrm{examined diagnosis pairs}}\times 100$
Consensus diagnosis	Agreement between multiple pathologists on a specific diagnosis for a study case; used as a ground truth diagnosis.
Equivalency	Tested by comparison of DM and LM separately to a gold standard (GS). DM versus GS is equivalent or superior to LM versus GS if the diagnostic performance is not significantly lower.
Diagnosis pair	Two diagnoses for the same case rendered at 2 different examination time points. Typically, LM versus DM diagnosis using the same pathologist.
Discordance	Disagreement between 2 study diagnoses. A validation study should define the type of discrepancy between 2 diagnoses (process, type, grade, secondary diagnosis, severity, terminology, etc) that comprises a discordant diagnosis. May be categorized as minor (eg, no clinical relevance) or major (eg, clinically relevant) discordance.
Gold standard (GS)	The best available method for rendering the “correct,” that is, ground truth, diagnosis. A true GS may not be available for histologic specimens.
Ground truth diagnosis	Best available estimation of the correct diagnosis using the GS method.
Kappa agreement	Level of reliability that is corrected for chance. The coefficient ranges between 0 and 1 (1 is the highest degree of reliability).
Noninferiority	The difference in the concordance rate between test modalities (DM vs LM) is not significantly more than is acceptable (defined by the noninferiority margin) as compared with the reference modality (LM vs LM).
Overall concordance rate (OCR)	$\mathrm{OCR}(\%)=\frac{\mathrm{concordant + minor discordant diagnoses}}{\mathrm{examined diagnosis pairs}}\times 100$
Referee pathologist	A pathologist that decides whether the diagnosis pairs from the study pathologist(s) are concordant or discordant.
Repeatability	Concordance rate for diagnosis pairs using the same viewing modality (LM vs LM or DM vs DM) by the same pathologist under the same conditions. Repeatability of LM is a suitable benchmark for a validation study.
Reproducibility	Concordance rate for diagnosis pairs using the same viewing modality (LM vs LM or DM vs DM) by different pathologists or under different conditions. This value may be used as an estimation for an acceptable diagnostic performance of LM versus DM.
Study pathologist	A pathologist that makes diagnoses from the study cases using LM and DM. They are blinded to the previously reported diagnoses and other study pathologists.
Validation of DM	A study with the goal of demonstrating and documenting acceptable performance (concordance rate, noninferiority, or at least equivalency) of the DM workflow for the intended application.
Washout period	Time gap between 2 examination time points (typically one with LM and one with DM) of the same case/slide read by the same pathologist in order to reduce recall of the previously rendered diagnosis.

Abbreviations: DM, digital microscopy; LM, light microscopy; #, number of.

### Outcome Measures

Validation studies typically evaluate the entire DM workflow as a whole by measuring the final outcome: the ability of pathologists to arrive at a “correct” diagnosis via visual assessment of WSI. Establishing whether DM is as reliable as LM for rendering a specific diagnosis is the primary goal. An optional secondary goal may be to assess whether the DM diagnosis is achieved efficiently or using appropriate image quality, which are not discussed in further detail in this review. Individual components of the DM workflow are generally not evaluated by outcome assessment. Therefore, a concluding questionnaire or discussions within the group involved in the validation study may be a valuable tool for obtaining user feedback. Possible outcome measurements of a validation study include the following:

- Diagnostic performance (primary measure)
  ˆ Concordance rate (intraobserver; at least between LM and DM)^56^
  ˆ Kappa agreement (intraobserver; at least between LM and DM)^17^
  ˆ Accuracy (compared to a gold standard)^20,56^
  ˆ Repeatability (intraobserver for the same modality)^17^
  ˆ Reproducibility (interobserver for the same modality)^58,59,62,70,74^
- Diagnostic confidence (primary measure)^17,28,31,42,71,76^
- Diagnostic time (secondary measure)^7,17,49^
- Image quality (secondary measure)^8,9,17,31,39^

*Diagnostic performance* between DM and LM is the most important outcome measurement. With extensive diagnostic experience, very high diagnostic performance may be achieved; however, difficulties may still arise from high case complexity or inappropriate tissue quality. Therefore, 100% concordance or a Kappa coefficient of 1.0 between LM and DM cannot be expected from a validation study. In veterinary pathology, Bertram et al^17^ demonstrated a lower overall concordance rate for round cell tumors (LM vs LM: 82.5% and LM vs DM: 80.0%) compared to epithelial and mesenchymal tumors (LM vs LM: 93.2% and LM vs DM: 91.4%) when only HE-stained slides were used (Suppl. Fig. S1). Needless to say, cases with a higher degree of difficulty/complexity will lead to an overall lower concordance rate. For 6 study pathologists examining the same round cell tumor cases, Bertram et al^17^ determined a concordance rate ranging between 80.0% and 92.5% using LM (LM vs LM; ie, a maximum difference of 12.5% between the 6 study participants), and ranging from 79.4% to 90.6% using DM compared to LM (ie, a maximum difference of 11.2% between the study participants; Suppl. Fig. S2). Measuring interrater concordance between these study pathologists would have resulted in a notable difference not attributable to the viewing modality. Consistent with our opinion, it is recommended to primarily measure intraobserver performance for validation of the DM workflow.^17,56^ The impact of the degree of familiarity with DM on diagnostic performance has not yet been examined.

The *concordance* rate (with 95% confidence interval) is used by almost all validation studies as the main outcome value. Based on the judgement of a referee pathologist, 2 diagnoses are considered concordant if they are the same and are considered discordant if there are notable discrepancies.^17,56^ The concordance rate is the number of congruent diagnosis pairs divided by all diagnosis pairs evaluated. As opposed to this 2-tier system, many studies utilize a 3-tier concordance outcome to include minor discordance and major discordance. The latter two are designated by whether or not the diagnostic discrepancy would lead to clinical or prognostic consequences or would alter patient management.^4,6,9,15,22,52^ In addition to the concordance rate, an overall concordance rate may be reported that includes both concordance and minor discordance rates, that is, all diagnoses that lead to the same clinical outcome or management.^12,63^ However, rigorousness of criteria when determining concordance/discordance is somewhat subjective and may vary between studies. We highly recommend defining what constitutes an agreement as well as the required extent of agreement between a diagnosis pair before a study is performed. Considerations for defining these parameters include the type of primary and possible secondary process or diseases, synonymous terminology, modifiers (such as severity, tumor grade, etc), lack of information in one diagnosis, and others. In previous studies, reference tables for synonymous terms and what constitutes minor and major discrepancies have been rarely utilized.^22,74^ Use of standardized diagnostic criteria and terminology or even a diagnosis checklist (as opposed to free text fields) may be helpful to facilitate comparison of paired diagnoses.^8,44,50,53,74^ Possible examples of discrepancies in tumor diagnoses are listed in Supplemental Table S2.

### Study Design

**Figures 2–4. fig2-03009858211040476:**
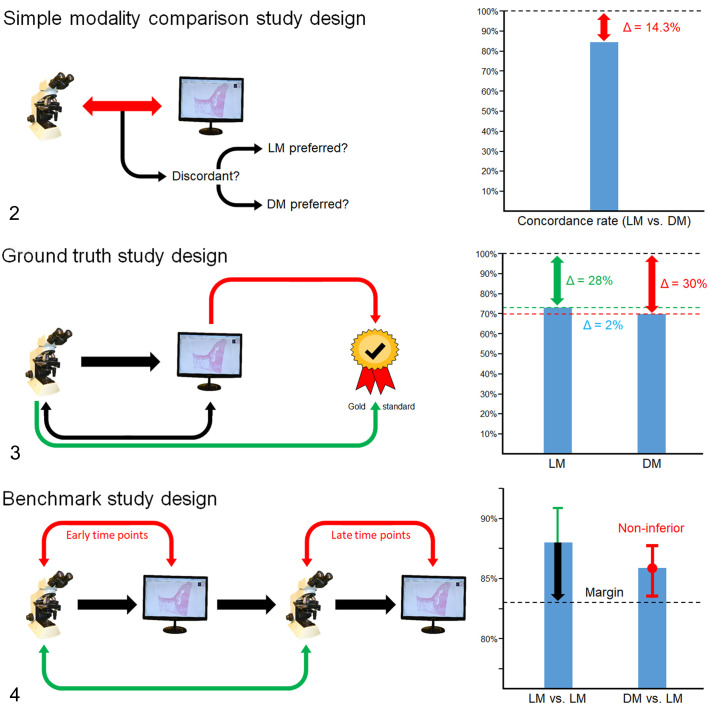
Comparison of different study designs. The diagrams depict the study course, data analysis and interpretation of the concordance rate of a simple modality comparison study (**Fig. 2**), ground truth study (**Fig. 3**), and benchmark study (**Fig. 4**). Raw data of the graphs are taken from previous studies.^17,20^ Δ, difference in the concordance rate.

We propose 3 major study design categories within which most published validation studies can be classified (Figs. 2 [Fig fig2-03009858211040476]–4). The overall objective as well as the tradeoff between time investment and validity of the results will guide the investigators in deciding which of the 3 study design categories should be used for their study (Table 2).

**Table 2.** Comparison of the 3 major validation study designs to compare digital microscopy (DM) and light microscopy (LM).

	Study design		
	Simple modality comparison	Ground truth	Benchmark
Attributes			
Study objective	Prove high concordance between DM and LM	Prove equivalency or superiority of DM compared to LM	Prove non-inferiority of DM vs LM
Examination time points	2	2 (+ ground truth)	3 or ideally 4
Time investment	+	++	+++
Case recall bias	Lower	Lower	Higher
Test modality	DM	DM and LM	DM vs LM
Reference modality	LM	Independent gold standard	LM vs LM (repeatability)
Gold standard dilemma	No/yes	Yes	No
Validity of results	+/++	++/+++	+++
Performance measurements and statistical tests			
Concordance rate	Yes	Yes	Yes
Kappa agreement	Yes	Yes	Yes
Accuracy	No	Yes	No
Fisher’s exact test	No	Yes	(Yes)
Noninferiority test	(Yes)	(Yes)	Yes

#### Simple Modality Comparison Studies

A simple modality comparison study measures the degree of concordance between DM and LM (Fig. 2). This study design is the simplest of the 3 with all cases being examined once with DM and once with LM at 2 examination time points (Suppl. Fig. S3). Diagnostic performance is measured by comparing DM (test modality) with LM (reference modality), which is calculated with one concordance rate value and/or κ agreement.^1–6,16,37,38,70^ The biggest limitation in the interpretation of discordant results between LM and DM is that no true reference value is determined. For example, using the same validation study methods, Al-Janabi et al^1,4^ determined 95% concordance in one study of gastrointestinal tract pathology and 87% concordance in another study of urinary system pathology. These concordance values are very high, but not perfect, as expected, and quite different. The results raise general questions: Is the value high enough? What is an acceptable cutoff value? Is the discrepancy between LM and DM caused by the limitations of DM? Is the first validation result better than the second?

Whereas reliable diagnostic performance certainly depends on appropriate DM workflow parameters, it is undeniably influenced by case complexity/difficulty and the pathologist’s skills regardless of the viewing modality.^17^ Visual assessment of microscopic slides depends on the perception of morphological patterns and their interpretation. This perception may possibly vary between and within the same pathologist depending on their diagnostic experience, professional opinion/mindset, and level of fatigue and concentration. There is not one concordance threshold that is appropriate for all studies. According to Pantanowitz et al,^56^ an acceptable (pass/fail) concordance rate is best determined by the good medical judgement of the pathologist and each investigator/laboratory has to decide for themselves whether the results of their validation are sufficient for their needs. Depending on methodological differences between validation studies, it may be extremely difficult to find a representative reference value from the literature. Snead et al^63^ used results from multidisciplinary team meetings in order to estimate a representative concordance rate and noninferiority threshold of 98% concordance, despite the lack of a study arm to determine a benchmark value for LM. The updated guideline from the College of American Pathologists emphasizes that laboratories should be especially alerted if the concordance rate is below 95%.^29^

The absence of a reference value is considered a major limitation of this study design and there are 2 possible methods of addressing this shortcoming to improve interpretability of validation study results. Both methods intend to estimate whether the DM viewing modality was more problematic in the cases examined. First, discordant cases can be reviewed by an expert or group of experts (usually using LM) in order to assess which diagnosis, LM- or DM-based, is preferred.^1–6,9,13,15,63^ With this approach, the suspected cause of error, such as insufficient digital image quality, can be discussed. Second, the intraobserver results can be compared to other study pathologists (interobserver reproducibility), if available, to estimate whether the viewing modality has a greater influence than the variance between pathologists.^58,59,62,70^

#### Ground Truth Studies

The goal of ground truth studies is to examine whether the diagnosis using LM or DM is equally consistent or even more (but not significantly less) often consistent with the “true” diagnosis (ground truth). Concordance between study diagnoses using LM or DM with the ground truth diagnosis is defined as accuracy.^56^ In addition to an examination time point for each case with LM and DM, there is also an independent ground truth diagnosis using a gold standard examination method (Suppl. Fig. S4). Thereby it can be evaluated how many “correct” or “incorrect” diagnoses were rendered with each viewing modality separately (Fig. 3). Differences in the percentage of correct diagnoses between LM and DM is mostly consistent with the influence of DM workflow parameters and can be statistically evaluated by a Fisher’s exact test^20,21^ or by a paired/2-sided noninferiority test (with a noninferiority margin of 4% or similar).^43^ For specific diagnostic tasks, such as identification of neoplastic processes, true positive (TP), true negative (TN), false positive (FP), and false negative (FN) can be defined and used for calculation of sensitivity $\left(\frac{\mathrm{TP}}{\mathrm{TP}+\mathrm{FN}}\right)$ , specificity $\left(\frac{\mathrm{TN}}{\mathrm{TN}+\mathrm{FP}}\right)$ , and accuracy $\left(\frac{\mathrm{TP}+\mathrm{TN}}{\mathrm{TP}+\mathrm{TN}+\mathrm{FP}+\mathrm{FN}}\right)$ .^20^

**Figure 5.**
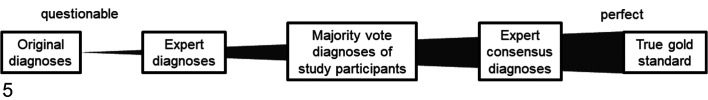
Proposed quality of different gold standard methods reported in validation studies for defining ground truth diagnoses.

A true gold standard, however, may not be available for all diagnostic pathology tasks. For example, a cytologic validation study may use histologic diagnoses or flow cytometric diagnoses (for lymphoid tumors) as a gold standard.^20,21^ Although histologic and cytologic specimens may not always allow the same interpretation, histology is independent to cytology (as it is not used by the study pathologists) and is generally considered superior (“true” diagnosis) for most disease entities (there are some exceptions). Therefore, it may be used as a gold standard (GS) method to determine “equivalency” or “superiority” (as opposed to agreement or concordance) between DM and LM. Although the concordance rates may be overall lower for both viewing modalities when comparing cytologic to histologic diagnoses, for the ground truth study design the difference of the concordance rates between DM versus GS and LM versus GS is relevant. In contrast, a gold standard for histologic specimens may be difficult to identify, which is the main limitation of this study design. Possibly special stains, immunohistochemistry or molecular methods may provide a superior diagnosis to histologic examination for some disease entities. Pathologist-derived interpretation of morphologic features in histologic sections is inherently subjective and not independent as they are based on the same specimen that is used for the study (usually glass slides). The histologic ground truth diagnosis has been previously defined as the original LM diagnosis obtained during routine diagnostic service,^25,29,49,52^ the diagnosis obtained from the most experienced pathologist,^23,53,73^ the majority vote (>50% pathologists agree) of the study pathologists using LM examination,^72^ or the consensus diagnosis of a group of experts.^28,43,71^ Ideally, the reference pathologists are not included among the study pathologists. While a majority vote is not independent (because in this situation the reference pathologists are the study pathologists), diagnosis by a single expert does not account for personal preferences or experience. Study pathologists are disqualified on the ground of bias. We consider a consensus diagnosis or a true gold standard to be the most advantageous (Fig. 5).

#### Benchmark Studies

The aim of this study design is to determine a benchmark concordance rate for LM itself as the benchmark viewing modality (LM vs LM; intraobserver LM repeatability) for the specific conditions of the study (Fig. 4). With this type of study, it can be determined how greatly the benchmark value is influenced by the sources of bias and a minimally acceptable concordance threshold for the test modality (DM vs LM) can be adjusted. Therefore, at least 3 examination time points are necessary (twice with LM and once with DM; Suppl. Fig. S5).^15,57,62^ Ideally, an additional fourth time point with a second DM examination is performed in order to reveal the size of the learning curve between the early (first and second) and late (third and fourth) time points (Suppl. Fig. S6).^17^ In addition, this strategy allows determination of intraobserver repeatability for DM (DM vs DM).^17,55^ However, calculation of the DM versus LM concordance rate for the combined early and late time points will result in a smaller confidence interval due to the higher number of diagnosis pairs, which can be accounted for by a somewhat smaller noninferiority margin (see below). This study design is similar to a crossover study; however, the study arms are longitudinal and not parallel.

With the obtained data, noninferiority can be evaluated with a 1-sided binomial test. In order to prove noninferiority, the null hypothesis (the measured discordance between DM and LM is greater than the noninferiority margin) must be rejected. This method evaluates whether the lower 2.5% margin of the 95% confidence interval of the test concordance rate is above an acceptable noninferiority threshold, which is obtained from lowering the benchmark concordance rate by a certain noninferiority margin (usually 4% or 5%, see Fig. 4).^15,17^ If the noninferiority margin lies within the 95% confidence interval of the test concordance rate, then the results are not noninferior. If the upper 2.5% boundary of the confidence interval of DM versus LM is below the noninferiority margin, then the results are significantly inferior to the benchmark modality. Similar to the noninferiority test, Shah et al^62^ used a 2-sided comparison of the 95% confidence interval (as opposed to using a noninferiority margin) of LM versus LM and DM versus LM. They interpreted no statistical difference if the 2 confidence intervals had any overlap. Other publications used a Fisher’s exact test determine the significance of the discrepancy rate^3^ or used a Wilcoxon signed-ranks test for comparison of κ agreement between the benchmark and test modality.^55^

The main limitation of this type of study design is the increased time investment due to the third and possibly fourth examination. Repeated evaluations of the same cases are associated with a higher recall bias that needs to be addressed with a sufficient washout time (interval of time between LM and DM examination of the same case) and possibly a randomized case order between examination time points in addition to suitable case numbers and scope (see below).

### Case Number and Scope

The breadth and number of cases deemed appropriate for the study design and intended use of DM are fundamental considerations for each validation study. These parameters represent a tradeoff between data validity and study feasibility (time investment, compliance of study participants, etc). According to the guidelines by the College of American Pathologists,^29,56^ a clinical validation study should encompass at least 60 cases for the main application and 20 additional cases for each supplemental application. In the current literature the case number varies significantly from >60^35,39,51,78^ to 3017 cases.^22,52,63,70^ Actual calculation of the required sample size using a 1-sided binomial test is rarely performed.^16,54,59,63^ Whereas the level of significance (0.05) is consistent, there is large variability between the power (70% to 99%) and the noninferiority margin (2% to 5%) that corresponds with a required sample size ranging from 100 and 3014 cases in previous studies.^13,16,54,59,63^ If small case numbers and multiple repeats are used (see benchmark study), it is important to reduce the recall bias as much as possible and/or allocate it equally between both viewing modalities. This is achieved via case re-identification, reordering between examination time points, long washout periods, and possibly absence of patient information.^17,44,52,57^

Whereas the multiple academic validation studies should eventually encompass all relevant subspecialties and tissue sources, clinical validation studies aim to reflect the broad spectrum of specimen types and diagnoses that are likely to be encountered during the intended use of DM.^56^ If multiple study participants are involved (which we certainly recommend), they can either examine the same or different cases. Examination of the same study case set will allow calculation of interobserver reproducibility (especially useful for simple modality comparison studies) and is easier for developing and conducting the study;^17,20,21,78^ however, it may possibly lead to amplification of the same errors. Alternatively, having different pathologists each examine a different subset of study cases will increase the total number of cases and thereby the scope/range and variance of the cases examined.^4,13,16,28,37,70^

The inclusion criteria of cases varies largely between published studies and certainly may influence outcome performance. Whereas many studies selected cases consecutively or randomly from a specific time period,^53,70^ others selected specific lesions based on the suspected prevalences.^16^ Some studies excluded cases with insufficient glass slide quality,^17^ unusual and difficult cases,^41^ or overly simple cases.^31^ Particularly for validation studies with relatively low case numbers, diagnostic performance of DM for uncommon cases is difficult to assess. In order to account for this, some studies have enriched their study set by adding cases that are generally difficult (such as round cell tumors) or presumably especially difficult for DM (such as cases with borderline malignancy).^17,52,68,74^ Selection of one key slide per case (using standard stains and excluding special stains) alleviates the time investment for study pathologists,^17,20,70^ but it may not accurately reflect the typical diagnostic setting.^16^ Additional considerations include whether study pathologists are able to request recuts, rescans (at higher resolution), additional sections, special stains, or immunohistochemistry,^22,25,45,63^ which might improve overall diagnostic performance. If no additional laboratory orders can be requested,^9,17,53,62^ it might be advisable to include a quality check of glass slides and WSI (focus, completeness of tissue, etc) before they are assigned to the study pathologists in order to ensure high-quality WSI for most appropriate diagnoses.^17,38,63^

### Course of Examination Time Points

For glass slides and WSI of the same case, study pathologists are usually blinded to previous diagnoses at different time points, separated by a washout period. Few studies have omitted the washout gap and compared their LM findings with the DM findings immediately after the DM diagnosis was obtained (side-by-side comparison).^45,68,76^ While this leads to a biased LM examination, this approach may be useful to identify differences in the visual perception of specific morphologic features between LM and DM.^45^ It may also be especially useful for continuing validation during diagnostic service after “going live.” However, most studies include two^4,5,20,21^ to four^17^ examination time points, depending on the study design, with a washout period of a few days^37^ to many months.^4,5^ For each time point the same “information” (same tissue sections, same staining, same patient information, etc) should be provided to the study pathologists. A sufficiently long washout period must be selected in order to ensure minimal case recall, which is especially relevant for benchmark studies. However, the washout period and the number of time points influences the overall duration of the validation study and might need to be reduced for the purposes of efficiency. An additional consideration is that the diagnostic criteria of an individual pathologist might change during an excessively long washout period.^10,56^ The College of American Pathologists^29,56^ recommend a washout period of at least 2 weeks. However, Campbell et al^26^ determined that a high percentage of the 120 slides examined were recalled after 2 weeks (40%) and even after 4 weeks (31%) by study pathologists and concluded that the recall rate was sufficiently high to cause significant bias.

In order to reduce the duration of the validation process, some studies used the original (LM or DM) examination from routine diagnostic work retrospectively as the first examination time point; thus, the same pathologist was only required to perform one additional examination with the other viewing modality.^4,6,15,72^ As an exception, few studies performed a “rapid validation” by only having one examination time point with DM (none with LM) and compared the diagnoses with a ground truth expert diagnosis.^23,39^ However, this method significantly reduces interpretability of the results.

Some studies have identified a learning effect when comparing early and late time points or phases of the study,^17,44,49,54^ which might be due to case recall, habituation with the study design (affects DM and LM), and/or increased familiarization with DM and the individual DM workstation. This suggests that the order in which LM and DM are used may influence this learning effect, although Pantanowitz et al^56^ did not find evidence for this. Nevertheless, a random order of the viewing modality and the study cases is recommended in the updated guidelines by the College of American Pathologists.^29^

If pathologists have not been familiarized with the DM workstation, a training phase prior to the study is highly recommended. Depending on the individual pathologist’s needs, the training phase can range from a single training slide^17^ to a training course of several days duration^54^ or review of hundreds of training WSI.^41^ If a learning effect cannot be excluded, an alternate method in which the pathologist views half of the study case set with each modality per examination time point might be useful.^17,43,49,70^

## What Has Been and Still Needs To Be Validated?

Since the early implementation of DM into pathology laboratories, there have been numerous validation study publications but few are from veterinary laboratories.^17,20,21^ This section summarizes the results from those studies for surgical pathology and cytology and includes a short review of the efforts faced in the field of toxicologic pathology.

### Human Surgical Pathology

The majority of the published validation studies in human pathology were performed with surgical pathology specimens. These publications show an overall high diagnostic performance of DM as compared to LM for many subspecialties such as dermatopathology,^5,8,13^ breast pathology,^6^ gastrointestinal pathology,^4,13^ and urogenital pathology.^1^ Recent large validation studies with case numbers >1000^13,22,52,63,70^ and systematic reviews have identified ample evidence for an overall reliable diagnostic performance of DM regardless of the scan magnification.^10,12,34,75^ Across 24 validation studies with a total of 19 468 DM-LM comparisons, Azam et al^12^ calculated an overall concordance rate of 98.3% (confidence interval: 97.4% to 98.9%) and complete concordance of 92% (confidence interval: 87.2% to 95.1%). Nevertheless, further validation studies have been requested especially for some subspecialties, such as hematopathology, ophthalmic pathology, or nephropathology.^10,12,34,60^ In addition, few studies have evaluated the performance of identifying specific morphologic features with WSI.^14,55,73^ A systematic review article on discordant diagnoses in published validation studies evaluating a total of 8069 diagnosis pairs revealed an overall discordance rate of 4%, for which the LM diagnosis had been favored over the DM diagnosis in 85% of the cases.^75^ The major sources of WSI-related discrepancy are classification of malignancy versus dysplasia, and detection/identification of small foci of disease and small objects such as microorganisms or nuclear details.^3,10,12,28,75^ Whereas 200× scan magnification had been sufficient for many diagnostic purposes,^8,25^ other cases in which more subtle morphologic features are important such as mitotic figures or microorganisms^3^ would benefit from higher image resolution. The same is true for z-stacking (WSI at multiple focus levels that allow fine focusing): most disorders (such as melanocytic lesions^68^) can be reliably diagnosed with WSI at a focal plane from thin tissue sections, whereas identification of microorganisms (such as *Helicobacter pylori* ^42^) may be improved by evaluating multiple focus planes.

### Human Cytopathology

DM of cytologic specimens is controversial^18^ and fewer validation studies are available compared to surgical pathology. For many cytologic specimens, low-resolution scans and lack of fine focus may hamper diagnostic performance and efficiency. In the authors’ experience, diagnostic applicability of DM largely depends on the method of specimen preparation. For example, 400× magnification and a single focus level may be appropriate for cytospin preparation of body fluids (such as bronchoalveolar lavage; based on our own experience subject to a validation study), while bone marrow aspirates may possibly require higher image resolution and multiple focus planes (z-stacks). For human cytology specimens, Girolami et al^33^ provide a systematic review of available validation studies (*N* = 19) and concluded that there is limited evidence for acceptable diagnostic concordance of DM and LM. Girolami et al^33^ criticized that only one validation study with human cytologic specimens^19^ used a benchmark study design. In our opinion, histology or flow cytometry of the same tissue lesion may offer a unique opportunity as a ground truth diagnosis to test for “superiority” (as opposed to “high concordance” or “noninferiority”) of the viewing modality for cytologic specimens.^20,21^ Although cytologic diagnosis and histologic diagnosis do not always match due to intrinsic differences in the diagnostic methods and a sampling bias,^69^ this discrepancy will affect both viewing modalities likewise and might therefore have a smaller bias (if LM and DM are compared independently to the ground truth; see ground truth study design) than the quality of an expert-derived, cytology-based ground truth diagnosis.

Regardless of the current challenges, technical advancements of the DM workflow and ongoing validation studies are considered to be the driving force for widespread implementation of DM for cytology in the future.^33^

### Veterinary Pathology

Publications of validation studies of whole-slide imaging from veterinary laboratories include one with surgical pathology specimens and two with cytologic specimens from dogs and cats (see below).^17,20,21^ Although the results of academic validation studies from human pathology may be extrapolated to veterinary pathology, further validation studies from a veterinary setting should be advocated, especially for specific applications within our field. Future studies should especially include interpretation and scoring of special stains (such as Ziehl-Neelsen stain) and immunohistochemical labeling.

Bertram et al^17^ examined diagnostic performance, diagnostic confidence, diagnostic time, and image quality of 80 canine surgical skin tumor biopsies in a benchmark validation study. Comparison of performance between DM (DM vs LM and DM vs DM) and LM (LM vs LM) revealed that diagnoses were noninferior for all tumor types combined and slightly lower (not noninferior) with DM for diagnosis of round cell tumors and grading of mast cell tumors. Higher scan resolution (0.25 µm per pixel compared to 0.5 µm per pixel) did not improve diagnostic performance. WSI of specimens stained with toluidine blue, that was used for differentiation of mast cell tumors from other round cells tumors, was perceived to have a less sufficient quality by the study pathologists than WSI of HE-stained specimens, which might have been influenced by the default scan settings.

The validation study by Bonsembiante et al^20^ evaluated 60 cytological specimens from dogs and cats. A true gold standard diagnosis was available for the included cases (ground truth study) and was obtained via histologic examination or flow cytometry for lymphoid tumors. The correct diagnosis (concordance with ground truth) varied for the 3 study participants between 65% and 73% for DM and between 65% and 78% for LM. No significant difference in diagnostic accuracy between the two viewing modalities was found.

For canine lymphoma, Bonsembiante et al^21^ validated intraobserver κ agreement and concordance between DM (400× magnification, z-stack) and LM for classification of cellular morphologic features and grade in 44 cytology specimens. The overall intraobserver κ agreement between DM and LM for numerous cytomorphologic features was fair to moderate (k = 0.34–0.52). Using flow cytometry as a reference (ground truth study), assessment of correct cytologic grade was not significantly different between DM and LM.

### Toxicologic Pathology

The use of DM for primary examination of toxicologic studies has generated great interest. However, there are only few published validation studies for toxicologic applications (in the preclinical environment).^23,40^ Long et al^46^ provide guidance on the technical aspects of validation of a whole-slide scanner system. In addition, a recent publication by Schumacher et al^61^ highlighted the steps necessary for achieving acceptance of digital pathology by regulatory authorities. Nevertheless, there is still some uncertainty about the minimum requirements for a validation study in a Good Laboratory Practice–regulated environment. Organizations must consider the complexity of the DM system and the risk DM has on patient safety and product quality.^46^ There are currently vigorous efforts from numerous toxicologic organizations, working groups and regulatory bodies to establish clear statements on the regulatory framework and develop guidelines on appropriate validation methods.^61^

## Conclusion

There is high methodological variation between published validation studies, each having advantages and limitations. The diagnostic concordance rate between DM and LM is the most relevant outcome measure, which is influenced (regardless of the viewing modality used) by different sources of bias including complexity of the cases examined, diagnostic experience of the study pathologists, and case recall. In Supplemental Figures S3–S6, we propose possible study courses for a simple modality comparison study, a ground truth study, and 2 benchmark studies (with 3 or 4 examination time points) that may be utilized for future validation studies. In the field of veterinary and toxicologic pathology, evidence for acceptable diagnostic concordance of DM is largely lacking and further validation study publications are needed, especially for specific applications in our fields.

## Supplemental Material

Supplemental Material, sj-pdf-1-vet-10.1177_03009858211040476 - Validation of digital microscopy: Review of validation methods and sources of bias Click here for additional data file. Supplemental Material, sj-pdf-1-vet-10.1177_03009858211040476 for Validation of digital microscopy: Review of validation methods and sources of bias by Christof A. Bertram, Nikolas Stathonikos, Taryn A. Donovan, Alexander Bartel, Andrea Fuchs-Baumgartinger, Karoline Lipnik, Paul J. van Diest, Federico Bonsembiante and Robert Klopfleisch in Veterinary Pathology
