# Supplementary material for: Validation of digital microscopy: Review of validation methods and sources of bias
Source: Vet Pathol. 2021 Aug 26;59(1):26–38. doi: 10.1177/03009858211040476 (PMC8761960; doi:10.1177/03009858211040476)
Supplement: Supplemental Material, sj-pdf-1-vet-10.1177_03009858211040476 - Validation of digital microscopy: Review of validation methods and sources of bias [file sj-pdf-1-vet-10.1177_03009858211040476.pdf]

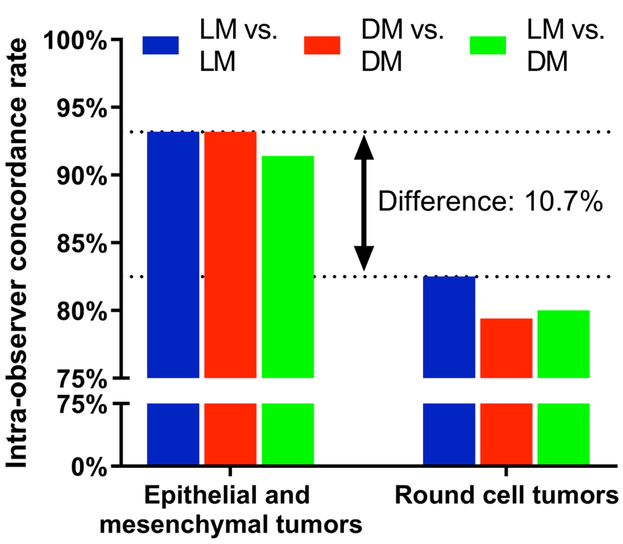

S1

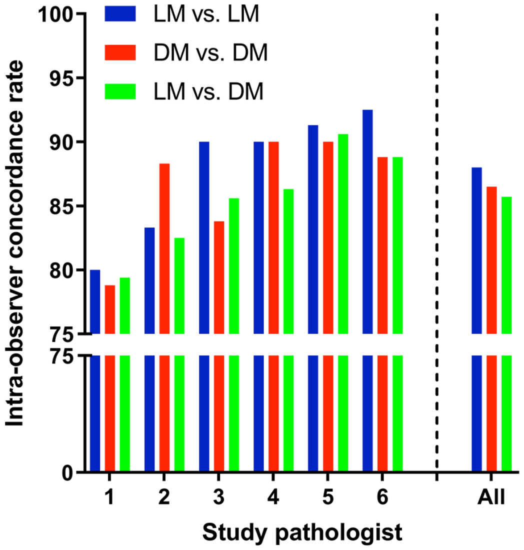

S2

Supplemental Figure S1 and S2. Examples of sources of bias for measuring concordance rate between light microscopy (LM) and digital microscopy (DM). **Figure S1.** Comparison of the diagnostic concordance rate between two tumor groups with different case complexity. Diagnosis of round cell tumor types clearly has a lower concordance rate regardless of the use of LM or DM. **Figure S2.** Comparison of the diagnostic concordance rate between six study pathologists for the diagnosis of different tumor types (sorted by rank). There is clearly a difference between individual pathologists regardless of the use of LM or DM.

Raw data of both figures is taken from the validation study by Bertram et al. (Bertram CA, Gurtner C, Dettwiler M, et al. Validation of digital microscopy compared with light microscopy for the diagnosis of canine cutaneous tumors. Vet Pathol. 2018;55: 490-500).

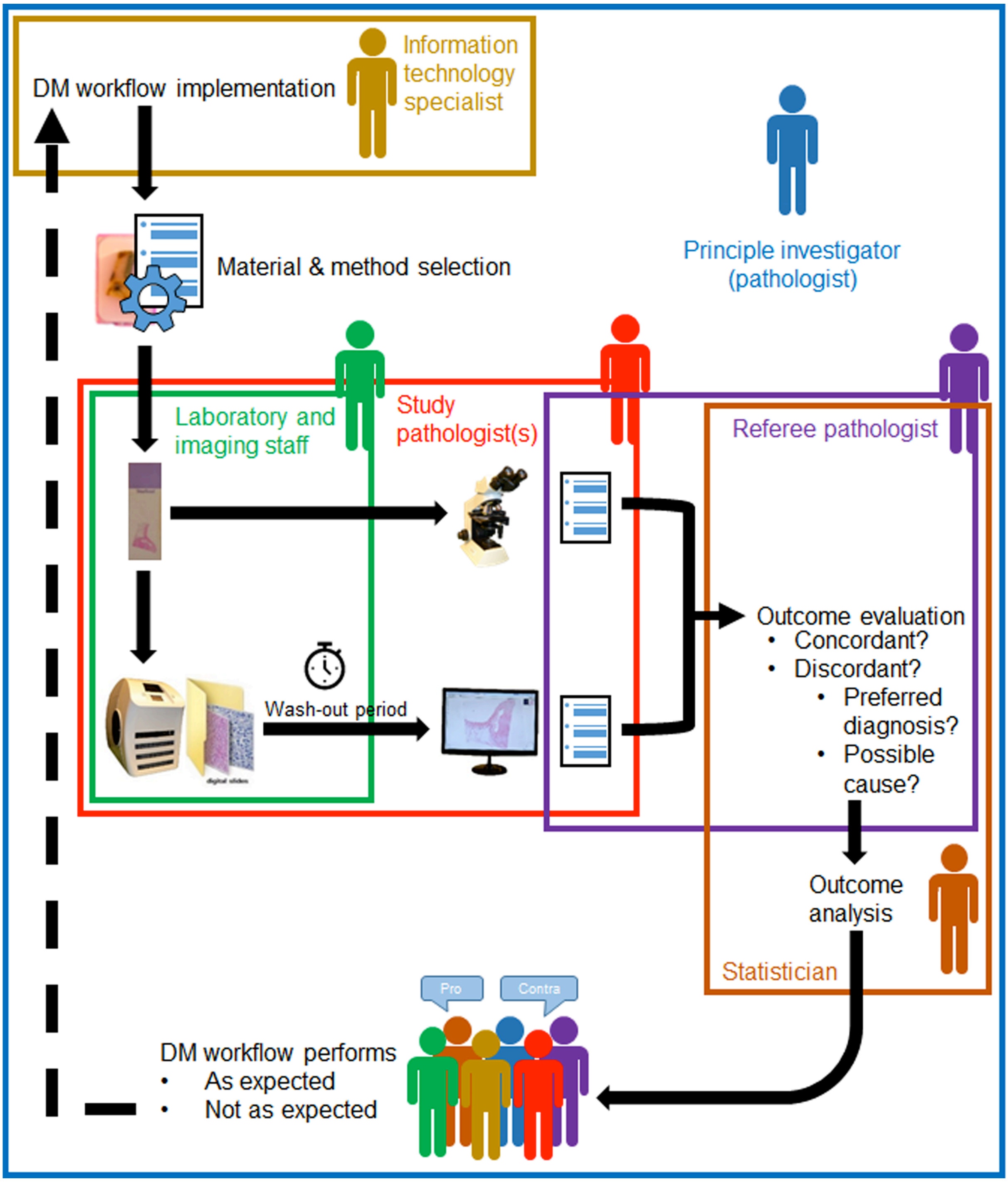

Supplemental Figure S3. Possible study course of a simple modality comparison study.

DM, digital microscopy

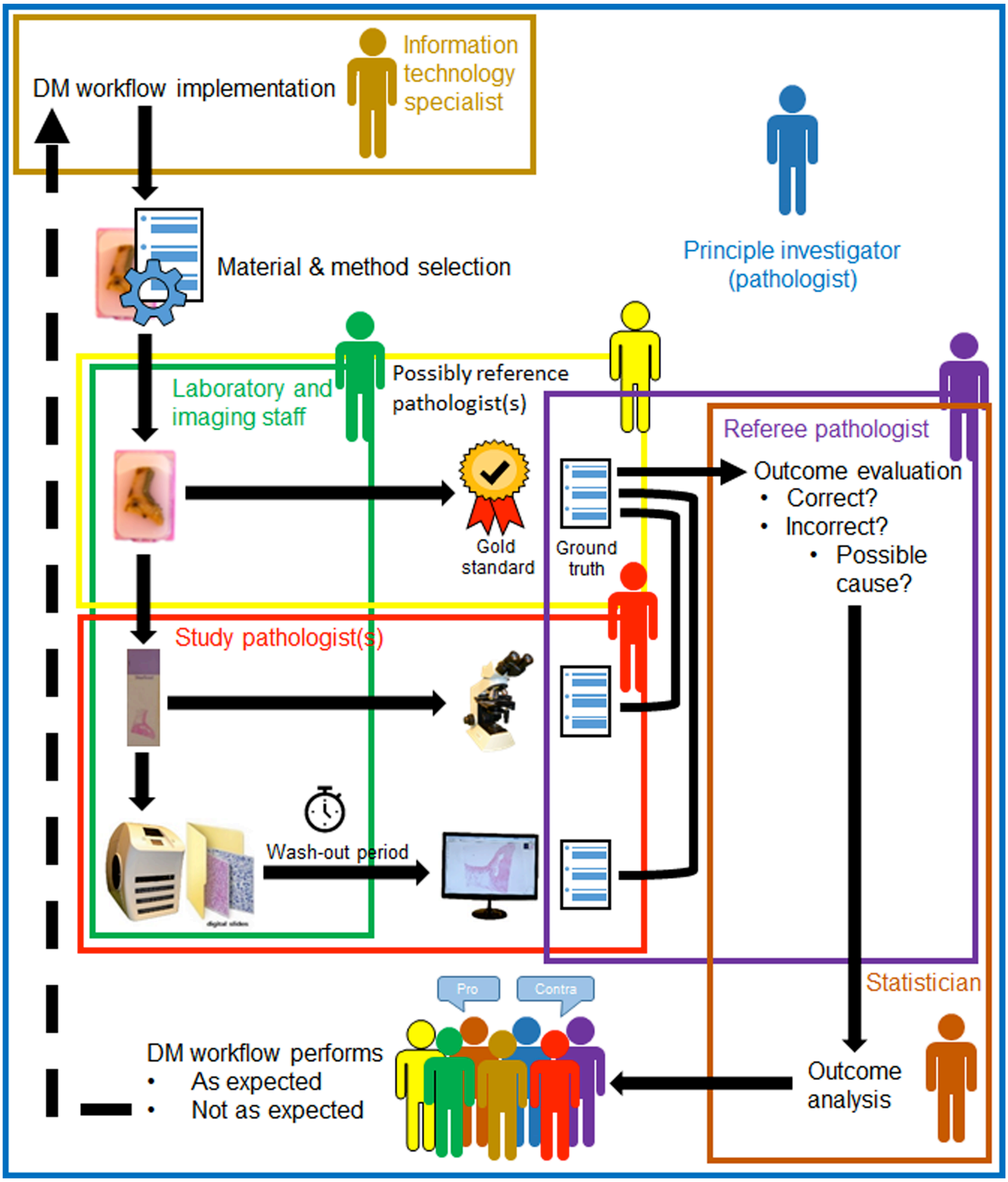

S4

Supplemental Figure S4. Possible study course of a ground truth study.

DM, digital microscopy

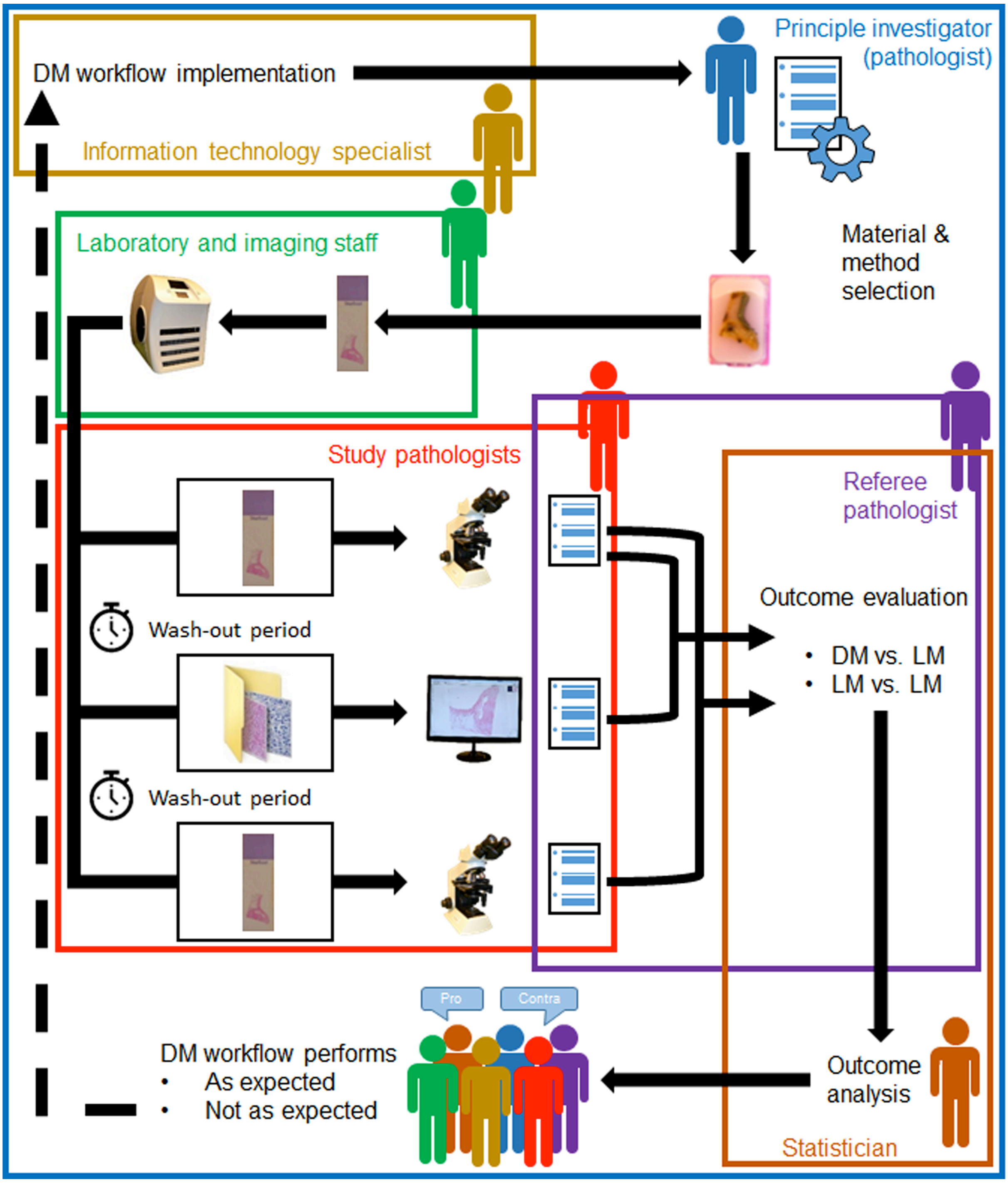

S5

Supplemental Figure S5. Possible study course of a benchmark study with three examination time points.

DM, digital microscopy; LM, light microscopy

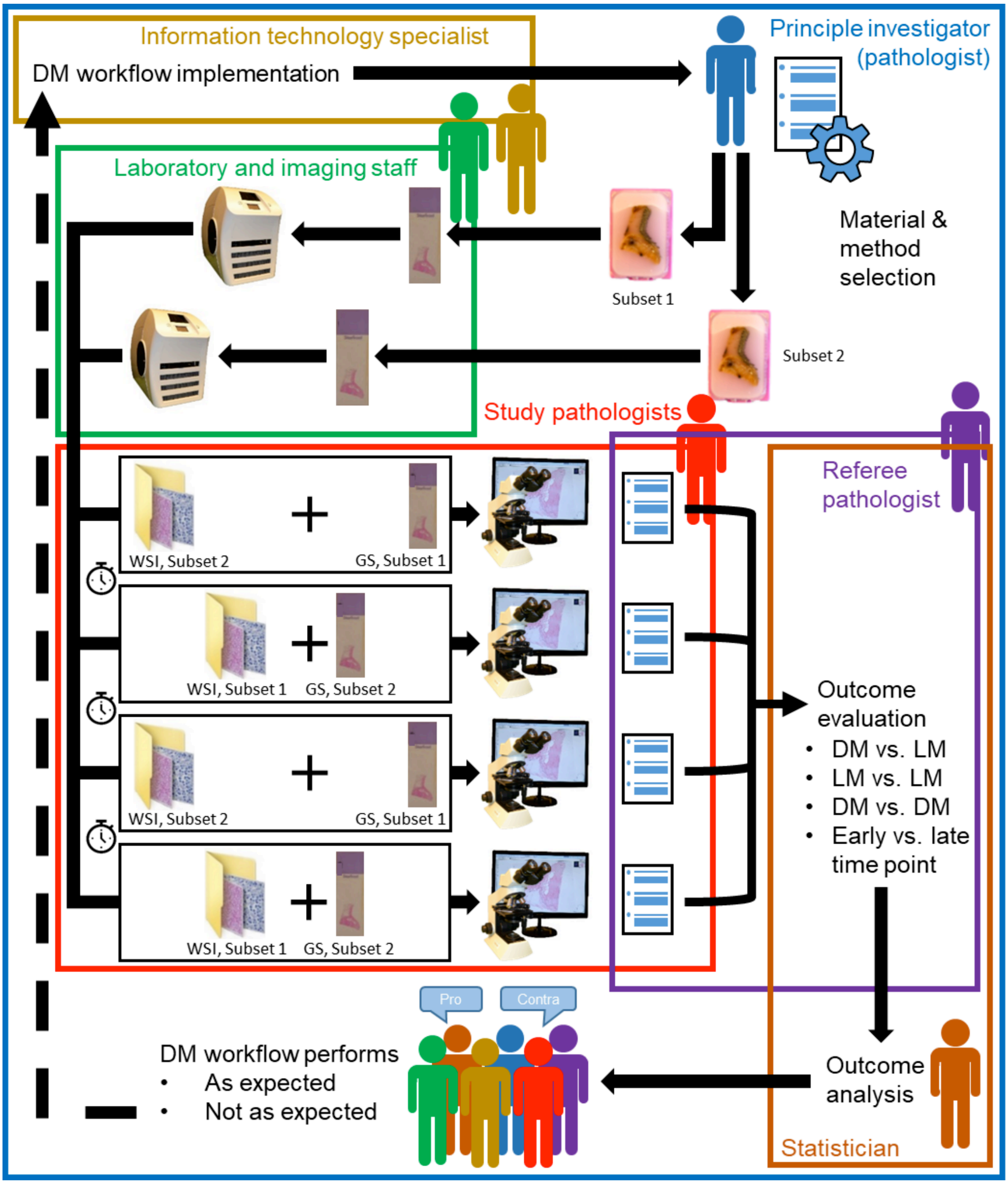

S6

Supplemental Figure S6. Possible study course of a benchmark study with four examination time points, randomized case order and division of the study cases into two subsets that are examined with light microscopy (LM) using glass slides (GS) or digital microscopy (DM) using whole-slide images (WSI) for each examination time point.

Supplemental Table S1. Summary of methods to consider for validation studies comparing DM to LM and recommendation by the guidelines for clinical validation studies from the College of American Pathologists (CAP) Pathology and Laboratory Quality Center (Reference: Pantanowitz L, Sinard JH, Henricks WH, et al. Validating whole-slide imaging for diagnostic purposes in pathology: guideline from the College of American Pathologists Pathology and Laboratory Quality Center. Arch Pathol Lab Med. 2013;137: 1710-1722).

Component of Materials and Methods	Options	Impact on interpretation of results and assessment of diagnostic performance	Recommendations by Pantanowitz et al. [a]
Study design	• Simple modality comparison study	• Lack of a suitable reference / benchmark for the concordance rate<br>• Review of discordant diagnosis	• "An acceptable (pass/fail) concordance rate for pathologists is best determined by the good medical judgment of the pathologist"<br>• Determine root cause of discrepancy
	• Ground truth study	• Evaluate LM and DM independently against a ground truth<br>• Fisher's exact test<br>• Quality of the gold standard method	• N/A
	• Benchmark study	• Availability of a benchmark (LM repeatability)<br>• Non-inferiority test<br>• Higher recall bias	• N/A

**Component of Materials and Methods**	**Options**	**Impact on interpretation of results and assessment of diagnostic performance**	**Recommendations by Pantanowitz et al.** [a]
Case numbers	• Estimate appropriate case numbers (tradeoff with time investment)<br>• Statistical calculation of required case numbers (1-sided binominal test)	• High relative influence of an individual discordant case in a study with small case numbers<br>• Small case numbers may not encompass the relevant breadth of lesions and image artifacts<br>• Different power and non-inferiority margin used for case number calculation	• At least 60 cases from one application and another 20 cases for each additional application
Case inclusion (specimen type, diagnoses, breadth)	• Retrospective or prospective selection of cases<br>• Inclusion (“enrich”) or exclusion of difficult lesions or specimens with poor quality<br>• One representative key slide per case vs. all slides per case (trade off with time investment etc.)<br>• Quality check of glass slides and whole-slide images prior to the study<br>• Availability/lack of special stains or immunohistochemistry	• Case complexity / difficulty<br>• Non-representative cases may not accurately reflect diagnostic performance of DM and LM<br>• “simple” cases may not reveal limitations of DM (possibly define and enrich with critical morphological features)	• Should be appropriate for and applicable to the intended clinical use (spectrum and complexity)<br>• Retrospective or prospective selecting of consecutive series of cases<br>• Avoid selecting only best cases<br>• Use all slides per cases that would be reviewed routinely<br>• Confirmation of complete tissue coverage of digital images

**Component of Materials and Methods**	**Options**	**Impact on interpretation of results and assessment of diagnostic performance**	**Recommendations by Pantanowitz et al.** [a]
Case information	• Availability or lack of patient information, clinical findings, radiographic findings, location of lesion etc.	• Recall of case information<br>• Case information may bias diagnosis (unrelated to microscopic findings)<br>• Availability of case information may improve concordance rate	• Same information for LM and DM examination<br>• Study should mimic the work environment in which the DM workflow will be used
Case order	• Case order consistent or randomized for different time points	• Increased possibility for recall bias if cases are not randomized	• N/A
Study pathologists	• Single pathologist<br>• Multiple pathologists examining the same cases (possibly amplification of same errors) or different study case set (increases total number of examined cases)	• Diagnostic experience/skills<br>• Experience with DM<br>• Experience with individual DM workstation<br>• Diagnostic diligence	• Include at least one pathologist who will be using the DM workflow<br>• Multiple study pathologists will lead to more robust and accurate results<br>• Adequately trained to use the DM technology<br>• Pathologists should be blinded to original diagnoses

**Component of Materials and Methods**	**Options**	**Impact on interpretation of results and assessment of diagnostic performance**	**Recommendations by Pantanowitz et al.** [a]
Case examination by study pathologists	• Free text answer or checklist / single or multiple choice<br>• (Non-)standardized diagnostic criteria and terminology<br>• Possibility of ordering rescans of glass slides (scan artifacts), new sections (tissue quality), special stains and immunohistochemical sections	• Predefined diagnosis list may need to be very complex or may restrict breadth of diagnoses<br>• Inconsistent diagnostic terminology may compromise determination of concordance / discordance<br>• Non-standardized diagnostic criteria may compromise ability to compare diagnoses between pathologists<br>• Rescan rate may represent the pathologist's ability to detect relevant scanning artifacts	• Study should mimic the work environment in which the DM workflow will be used
Course of study	• 2-4 examination time points (with wash-out)<br>• Side-by-side comparison (no wash-out)	• Recall bias (length of washout period)<br>• Learning curve for DM	• Minimum of two week washout period between DM and LM
Viewing modality order	• First LM, then DM<br>• First DM, then LM<br>• Order of DM and LM randomized per study pathologist<br>• Use LM and DM for different study cases (randomized) per each examination time point	• Recall bias<br>• Learning curve for DM (and LM with little prior diagnostic experience)	• Random or non-random order can be chosen

Component of Materials and Methods	Options	Impact on interpretation of results and assessment of diagnostic performance	Recommendations by Pantanowitz et al. [a]
Primary outcome measures	Diagnostic performance:<br>• Concordance rate<br>• Overall concordance rate<br>• Kappa agreement<br>• Accuracy<br><br>• Repeatability<br>• Reproducibility	• Availability of a suitable reference value/benchmark (ideally gold standard diagnosis or LM repeatability; possibly inter-observer reproducibility)	• Intra-observer concordance rate (LM vs. DM)<br>• An acceptable (pass/fail) concordance rate is based on a pathologist's good medical judgment<br>• Determine root cause of discrepancy
	• Diagnostic confidence	• Subjective measure (pathologist's attitude towards DM and LM)<br>• Discrete scores that may not detect small differences in small case sets	• N/A
Secondary outcome measures	• Diagnostic time	• Individual workflow and workstation	• N/A
	• Score on whole-slide image quality	• Subjective measure (personal preferences)<br>• Scan parameters	• N/A
Performance judgement (by referee pathologist)	• Concordance vs. discordance (2-tier)<br>• Concordance vs. minor discordance vs. major discordance (3-tier based on clinical relevance or outcome)	• Rigorousness of decision criteria when determining concordance or discordance of diagnoses pairs<br>• Use of a predefined or decision criteria and terminology such as a checklist	• Determine root cause of discrepant cases

Abbreviations: DM Digital microscopy, LM light microscopy, N/A not available

Supplemental Table S2. Comparison of possible concordance versus discordance decision thresholds for two diagnoses with 2-tier, approximate (exaggeratedly soft) comparison, a 2-tier, point-by-point comparison or a 3-tier system based on clinical relevance of possible discrepancies.

**Diagnosis 1**	**Diagnosis 2**	**Concordance/discordance decision**			**Decision criteria**
		**2-tier, approximate**	**2 tier, point-by-point**	**3-tier based on clinical relevance**	
Fibrosarcoma	Fibrosarcoma	Concordance	Concordance	Concordance	Same disease entity
Soft tissue sarcoma	Soft tissue sarcoma	Concordance	Concordance	Concordance	Same disease subgroup, entity not specified
Soft tissue sarcoma	Soft tissue spindle cell tumor	Concordance	Discordance	Minor discordance	Likely same entity, but different terminology
Fibrosarcoma	Soft tissue sarcoma	Concordance	Discordance	Minor discordance	Different specificity of disease name
Fibrosarcoma	Leiomyosarcoma	Concordance	Discordance	Minor discordance	Different entities, but same disease subgroup (i.e. soft tissue sarcoma)
Fibrosarcoma	Mast cell tumor	Discordance	Discordance	Major discordance	Different disease entities and subgroup
Fibrosarcoma	Fibroma	Discordance	Discordance	Major discordance	Different malignancy
Fibrosarcoma	Fibrosarcoma Grade III, surgical margins: incomplete	Concordance	Discordance	Major discordance	Same entity, lack of information on primary disease
Fibrosarcoma, hyperkeratosis of epidermis	Fibrosarcoma, ulceration of epidermis	Concordance	Discordance	Minor discordance	Same main disease entity, different secondary disease
Cutaneous Fibrosarcoma Grade II	Subcutaneous Fibrosarcoma Grade III	Concordance	Discordance	Major discordance	Same disease entity, but different modifiers
